# Supervisors’ Academic Supervising Behaviors and Graduate Students’ Academic Thriving: The Mediating Roles of Admiration and Task Engagement

**DOI:** 10.3390/bs15060754

**Published:** 2025-05-30

**Authors:** Yang Wu, Zhenzhen Chen, Dongjing Zhang

**Affiliations:** 1 School of Marxism, Huazhong University of Science and Technology, Wuhan 430074, China; 2 School of Psychology & Mental Health Center, Central China Normal University, Wuhan 430079, China; chenzhenzhen@mail.ccnu.edu.cn

**Keywords:** supervisor’s academic supervising behaviors, admiration, task engagement, academic thriving

## Abstract

This study aims to examine the association between supervisors’ academic supervising behaviors and academic thriving of graduate students, as well as its underlying mechanisms—the mediating effects of admiration and task engagement. A sample of 1792 graduate students in central China was recruited to participate in this study (52.3% male; *M*_age_ = 23.266, *SD*_age_ = 5.435). The results show that the supervisors’ academic supervising behaviors are positively associated with graduate students’ self-reported academic thriving; furthermore, the mediating effects of both admiration and task engagement in the relationship are also significant, which consist of three mediating paths—the separate mediating effects of admiration and of task engagement, and serial mediation involving both admiration and task engagement. This study found that frequent supervising behaviors may promote academic thriving in graduate students through increasing students’ admiration for supervisors and task engagement. The findings not only contribute to our understanding of graduate students’ academic thriving and its mechanisms, but also add to the literature on promoting a positive psychological state of graduate students as well as graduate education.

## 1. Introduction

With the rapid development of postgraduate education in the world, the number of graduate students continues to increase, and multiple issues involving graduate education have received widespread attention, such as their mental health problems (Chen et al., 2023; T. M. Evans et al., 2018; Murguía Burton & Cao, 2022; Satinsky et al., 2021) and career development (Feldon et al., 2019; Wang et al., 2019; Way et al., 2019). Graduate students’ academic adaptation has been extensively examined, due to its important role in their mental health, interpersonal relationships, and academic career (Feldon et al., 2019; Li et al., 2024; Murguía Burton & Cao, 2022; Way et al., 2019). As a positive indicator of academic adaptation, academic thriving refers to a combined sense of growth and vitality (Kern & Sun, 2020), and can predict better life satisfaction and higher academic performance (e.g., Green & Rizwan, 2024). Given its importance, academic thriving has been the focus of recent studies and needs further examination.

Among the numerous factors influencing graduate students’ overall adaptation and academic thriving, students’ relationship with supervisors featured prominently in the research literature (Lienard et al., 2018; Sun et al., 2024). Although slight differences exist, in the present study, we use the words “mentor”, “advisor”, and “supervisor” interchangeably to refer to the staff member in charge of a whole range of educational tasks for the graduate students, such as academic guidance, support, and supervision. Most recent studies have examined the nature and deleterious effects of improper supervisor behaviors, such as abuse, sexual harassment (Tenbrunsel et al., 2019), academic misconduct (Johnson & Huwe, 2002), or various subtle stresses caused by supervisors (Wu et al., 2023; Wu et al., 2022). The growing research attention to the improper behavior of supervisors can potentially help address issues that may cause adverse outcomes for graduate students. However, to improve the health and adaptation of graduate students, the positive side of supervisors’ behaviors also warrants empirical consideration. The present study, therefore, aims to focus on whether graduate students could benefit from the positive behaviors of supervisors and thrive during their graduate studies.

### 1.1. Thriving for Graduate Students and Its Antecedents

As a key concept in positive psychology, thriving has garnered increasing research attention in recent years (Green & Rizwan, 2024; Kern & Sun, 2020; Y. Liang et al., 2024; Schreiner, 2013). Though definitions for thriving abound (Brown et al., 2017), a common theme across all the definitions is that thriving is a desired state as envisaged under humanistic doctrines and pertains to optimal human functioning (Brown et al., 2017; Epel et al., 1998; Spreitzer et al., 2005). Two broad characteristics of a typical thriving individual would be success at coping with challenges and a sense of vitality or elation from feeling growth (Kern & Sun, 2020; Spreitzer et al., 2005); thus, thriving can be defined as “the joint experience of development and success” (Brown et al., 2017).

The researchers may approach thriving from diverse perspectives, viewing it either as a global concept or as a domain-specific one (Brown et al., 2017). For example, separate lines of research have examined domain-specific thriving in work contexts (Kleine et al., 2019; Spreitzer et al., 2005) and in educational settings (Gerson & Fernandez, 2013; Schreiner, 2013), aside from others that mainly consider it as an overall state of mental health (Kern & Sun, 2020). As a positive psychological state, thriving can predict higher performance, life satisfaction, and subjective well-being at work or college (Porath et al., 2012; Walumbwa et al., 2018; Zhao et al., 2024), and in specific task domains, thriving can drive innovation and creativity (Sahadev et al., 2024; Shen et al., 2024; Zhan et al., 2025). For graduate students, academic performance (success, failure, struggle, stagnation, etc.) has a significant impact on their mental health and well-being—perhaps even more so than on undergraduates (cf. Schreiner, 2010, 2013). The academic aspects of graduate studies dictate that students not only take courses in the traditional sense, but also conduct research or serve as research apprentices under the guidance of their supervisors, to develop necessary academic skills (Lienard et al., 2018; Roach et al., 2019). Therefore, whether graduate students can effectively cope with challenges that arise during research, grow academically, and feel energetic and self-efficacious as a result—hallmarks of graduate students’ academic thriving—can be a good indicator of their overall functioning at graduate school. To date, there is scant research on the academic thriving of graduate students, and the present study aims to explore its influencing factors and underlying mechanisms.

### 1.2. Supervisors’ Academic Supervising Behaviors and Graduate Students’ Academic Thriving

Thriving can be influenced by individual-, group-, and sociocultural-level factors (Kern & Sun, 2020). Past research has documented that group support (Paterson et al., 2014), trust (Y. Liang et al., 2024), psychological safety (Frazier & Tupper, 2018; Zhan et al., 2025), mutual help and information sharing (Frazier & Tupper, 2018), as well as individual discretion given in the task environment (Spreitzer et al., 2005), can all be conducive to task-related thriving (Niessen et al., 2012). For graduate students, supervisors often assume multiple roles as mentors, team administrators, and research collaborators, all of which can greatly influence the immediate academic atmosphere that surrounds students. This may, in turn, affect students’ mental health and academic thriving. For example, supervisor support is positively associated with graduate students’ psychological health (Nielsen et al., 2017).

Supervisor’s academic supervising behaviors refer to an inclusive set of behaviors that assist, guide, monitor, and evaluate students as they progress during graduate education, with the aim of elevating their academic capabilities and honing their research skills (Peng, 2019; Roach et al., 2019). This often involves, for example, urging students to focus on main research targets, giving timely feedback or help, and participating in academic discussions. Graduate students can benefit from these behaviors by being enabled to zero in on their research topic and position themselves in the research literature relatively quickly (Feldon et al., 2019; van de Pol et al., 2010), to implement their research more efficiently via intellectual synthesis with supervisor (Lienard et al., 2018), and to be more aware of their academic advancement and growth (C. Evans, 2013). As a result, students may be more likely to experience growth and a sense of vitality during the process (Kunter et al., 2008; Nozaki & Mikolajczak, 2020). Based on this, it was hypothesized that academic supervising behaviors from supervisors would be positively associated with graduate students’ academic thriving (H1).

### 1.3. Mediating Role of Task Engagement

According to the socially embedded model of thriving at work (Istingtyas et al., 2025; Spreitzer et al., 2005), thriving is the psychological consequence of agentic work behaviors (e.g., exploration, engagement, or actively relating with coworkers), which are the results of two types of work-related variables, i.e., unit contextual characteristics (e.g., decision-making discretion and climate of trust) and resources produced in the action of work (e.g., knowledge, positive meaning, positive affect resources, and relational resources). As a proximal predictor of thriving and typical “agentic work behavior”, task engagement features prominently in multiple models for thriving (Brown et al., 2017; Kleine et al., 2019; Spreitzer et al., 2005).

Task engagement refers to the extent to which individuals focus on the task and devote themselves to the task behaviorally and emotionally, and is generally considered a critical link between contextual factors (e.g., unit characteristics, and resources) and individuals’ thriving (Spreitzer et al., 2005; Yang et al., 2024). One example could be the situation where task demand and boundaries are set and resources are amply provided such that the participants would perceive the task as challenging but achievable (e.g., Keller & Bless, 2008). Here, the contextual factors may engender higher task engagement, which, in turn, causes growth-related experiences (e.g., flow experience or thriving). Moreover, being highly engaged or involved in a task may lead to a more proactive approach to tasks, become more likely to identify shortcomings and devise innovative solutions, and elevate one’s willingness to acquire task-related skills (Paterson et al., 2014; Rothbard, 2001; Sahadev et al., 2024). Ultimately, it may help elicit an experience of growth and thriving (Spreitzer et al., 2005). On the other hand, higher task engagement can also increase the likelihood of success as well as individuals’ efficacy to overcome challenges (Bandura, 1977), which in turn fosters thriving. Among graduate students, academic task engagement can predict better academic skill acquisition (Feldon et al., 2019) and higher self-efficacy (Green & Rizwan, 2024), both of which can contribute to academic thriving. Thus, task engagement would be positively associated with their academic thriving.

Despite the well-documented link between task engagement and thriving (Y. Liang et al., 2024; Liu et al., 2021), it has not been replicated in the graduate contexts, in which context-specific antecedents to task engagement are yet to be tested. For graduate students, the academic supervising behaviors of the supervisors can be a vital contextual factor associated with students’ academic engagement. According to the integrative model of human growth at work, supervisory feedback can help dispel uncertainty about work and set goal priorities for employees, and can thus serve as a contextual enabler for task engagement and thriving (Spreitzer & Porath, 2014). Supervisors can help evaluate students’ progress, identify their potential, and set proper tasks with medium-level difficulty for graduate students; supervisors may also instill a positive meaning in the task, and perceiving the task as meaningful can inspire motivation (Brown et al., 2017; Fishbach & Woolley, 2022). Both can cause higher engagement. Relatedly, a few studies indeed showed that supervisors’ academic support and effective leadership could facilitate graduate students’ active engagement (McWilliam, 2009; Nielsen et al., 2017), which is a precursor to thriving. Therefore, it was hypothesized that the academic supervising behaviors of the supervisors could positively predict graduate students’ academic thriving through the mediating effect of task engagement (H2).

### 1.4. Mediating Role of Admiration

In the theoretical frameworks following the tradition of the self-determination theory (e.g., socially embedded model of thriving at work), individual thriving is usually considered as occurring and self-sustaining through a “positive spiral” (Goh et al., 2021, p. 198) involving contextual factors and individual’s emotional and behavioral interactions with them. Positive affect is a vital component within this context-to-individual link, and is itself a major predictor of thriving (Liu et al., 2021) in the socially embedded model of thriving at work.

Admiration is a ubiquitous positive emotion “elicited by virtue or skill above standards” (Onu et al., 2016) of others. It is often a mixture of the acknowledgment of others’ excellence and a sense of wonder or awe (Algoe & Haidt, 2009; White et al., 2024). Experiencing admiration may galvanize an individual, elevate their sense of efficacy, and thus generate an energizing experience (Schindler et al., 2015), all of which can bring benefits to individual psychological well-being (Pan, 2024) and help mitigate intergroup conflicts (Čehajić-Clancy et al., 2024). In addition, competence, role prototypicality, and attainability are considered important elicitors of admiration (Onu et al., 2016). Specifically, the evidence of competence in a person that is prototypical of his or her role and attainable for others—should they intend to emulate—would elicit admiration for the person. For graduate students, competent and responsible supervisors can be potential role models due to their academic achievements and other attributes, which are often evident in their supervising behaviors; and the subsequent admiration and aspiration to learn from them may foster higher academic thriving. Past research has often treated supervision and positive affect as predictors parallel of thriving (Istingtyas et al., 2025; Liu et al., 2021) and does not consider the possible mediation pathway. Based on these factors, it was hypothesized that admiration for supervisors can mediate the link between supervising behaviors and academic thriving (H3).

Moreover, admiration for role models may lead to an identification with values and beliefs espoused by the admired role model (Schindler, 2024), and further induce a tendency to emulate the admired role model (Čehajić-Clancy et al., 2024; Lockwood & Kunda, 1999; Marx et al., 2009; Schnall et al., 2010), potentially eliciting more “agentic work behaviors”, including task focus, task engagement, and exploration (Brown et al., 2017; Spreitzer et al., 2005). For example, in organizational contexts, admiration can engender organizational commitment and organizational citizenship behaviors (Thomson & Siegel, 2013; Vianello et al., 2010) and further improve employees’ task engagement (Cesário & Chambel, 2017; Gupta et al., 2016). As scholars themselves, graduate supervisors are often students’ first point of contact with academia and have the potential to shape their perception of its values and norms. Their dedication to mentoring and supervision may elicit admiration from students, reinforcing their identification with academic ideals and fostering greater academic engagement and, ultimately, academic thriving. So far, no previous studies have directly tested the possible serial mediation of admiration (a type of positive affect) and task engagement (a type of agentic work behavior) in the link between supervisory behaviors and thriving. Based on this, the present study hypothesized that admiration for supervisors and engagement can serially mediate the link between supervising behaviors and academic thriving (H4).

## 2. Methods

To explore the mediational mechanism, we adopted a cross-sectional design and collected data using questionnaires from graduate students in two research universities. Latent mediation models were then employed to test the four hypotheses. This approach enables the efficient collection of data, and the use of latent models is especially suitable for the estimation of mediational paths while controlling for measurement errors.

### 2.1. Participants

A convenient sample of 2100 graduate students from two research universities in central China was recruited to participate in this study. As shown in Table 1, excluding incomplete or careless responses (for exclusion criteria, see Procedure Section), the final sample consisted of 1792 participants (valid percentage: 83.333%), of which 937 were male (52.288%) and 855 were female (47.712%). The average age of participants was 23.266 years (*SD* = 5.435). Among the participants, there were 135 PhD candidates and 1657 Masters students; 396 of them were majoring in the disciplines of humanities and social sciences, and 1396 of them in disciplines of science, engineering, agriculture or medicine. The titles of their supervisors were Junior/Assistant Lecturer (2), Lecturer/Assistant Professor (103), Associate Professor (596), Professor (1090), and Others (1). The age of the supervisors was categorized into lower than 30 yrs. (38), 31–40 yrs. (702), 41–50 yrs. (607), 51–60 yrs. (378), and higher than 60 yrs. (67).

### 2.2. Measures

#### 2.2.1. Supervisors’ Academic Supervising Behaviors

Items of supervisors’ academic supervising behaviors were first developed via an open-ended questionnaire in a separate sample of graduate students (*N* = 133 with 99 males), and the initial 10 items were then screened in a focus group discussion consisting of research assistants and graduate students, which resulted in the final 7 types of academic supervising behaviors. A 7-item scale was then developed based on the seven behaviors (e.g., help determine research topic, and participate in discussions over specific research details). Participants rated their supervisors on the frequency of each supervising behavior on a 5-point Likert scale, ranging from 1 (*never*) to 5 (*frequent*). A confirmatory factor analysis indicates good validity, S-B χ^2^(14) = 165.854, RMSEA = 0.078, CFI = 0.959, TLI = 0.938, with standardized loadings of each item ranging from 0.586 to 0.796; in addition, the Cronbach α is 0.879.

#### 2.2.2. Admiration

The 4-item subscale for admiration from the Admiration and Adoration Scale (ADMANDOS, Schindler et al., 2015) was employed to measure the graduate students’ admiration for their supervisors. In the present study, we translated the items into Chinese and slightly adapted the items such that the target became the supervisor (e.g., I feel that my supervisor’s ability or behavior is admirable). A confirmatory factor analysis indicated good validity, S-B χ^2^(2) = 18.916, RMSEA = 0.075, CFI = 0.992, TLI = 0.975, with standardized loadings of each item ranging from 0.834 to 0.944; in addition, the Cronbach α is 0.934.

#### 2.2.3. Academic Thriving

We adapted the 10-item Thriving at Work scale (Y. Han & Wei, 2013; Porath et al., 2012) to measure graduate students’ feeling of thriving during their study and academic work while being graduate students. The scale has two dimensions, Learning and Vitality, and each consists of five items. Example items are “… I continue to learn more as time goes by” (Learning) and “… I feel alive and vital” (Vitality). All items were initially started with “At work” and were adapted in the present study to start with “While being a graduate student”. Each dimension has one negatively worded item, and following the past research, its method variance should be treated before modeling (Weijters et al., 2013; Wu et al., 2017). Participants rated their agreement with the items on a 7-point Likert scale (1 = *strongly disagree*; 7 = *strongly agree*). The scale had good reliability (Cronbach α = 0.940 for overall thriving) in the past research (Porath et al., 2012). A confirmatory factor analysis indicated good validity, S-B χ^2^(33) = 218.056, RMSEA = 0.056, CFI = 0.968, TLI = 0.956, with standardized loadings of each item ranging from 0.834 to 0.944; in addition, the Cronbach α is 0.846 (overall), and for each dimension, 0.754 (Learning) and 0.736 (Vitality).

#### 2.2.4. Task Engagement

Following the past research (Niessen et al., 2012; Rothbard, 2001), the present study used four items adapted from relevant dimensions of the Discipline Commitment scale (C. Xu, 2013) to measure graduate students’ engagement with their study task. Example items include “I enjoy reading books relevant to my discipline” and “I will get excited when studying relevant courses”. Items were rated on a 7-point Likert scale ranging from 1 (*strongly disagree*) to 7 (*strongly agree*). A confirmatory factor analysis indicated good validity, S-B χ^2^(2) = 13.653, RMSEA = 0.057, CFI = 0.994, TLI = 0.983, with standardized loadings of each item ranging from 0.783 to 0.847; in addition, the Cronbach α is 0.887.

### 2.3. Procedure

Participants were recruited from two large research universities in Central China, as research universities have a higher percentage of graduate students than undergraduate students and more comparable supervisor–student relationship patterns than smaller universities or research institutions. Participants filled out the questionnaire in an electronic form via classroom invitation or email. Participants were asked to complete the questionnaire alone, in a quiet place, and in a single, uninterrupted session of their choice within 1 week of acceptance. Those who successfully finished the questionnaire would receive course credits or a small gift worth approximately CNY 5.

To identify possible careless responders (Curran, 2016), we inserted an intentionally bogus item (Ward & Meade, 2023), “I have visited every country in the world”, in the questionnaire. On a 7-point Likert scale (1 = *strongly disagree*; 7 = *strongly agree*), any responses that showed agreement (i.e., greater than 1) were identified as careless responses. Moreover, incomplete responders were those who did not finish the questionnaire. We excluded both types of responders from further analysis.

### 2.4. Statistical Analysis

For descriptive statistics and correlations based on observed variables, data were prepared using IBM SPSS 22.0. To better accommodate the measurement errors, data were further modeled using Mplus 8.0 with a robust maximum likelihood estimator to explore the factor structures of each scale (using confirmatory factor analysis) and the latent correlations among key variables. A latent mediation model was specified to accommodate measurement errors and to increase the estimation accuracy (Gonzalez & MacKinnon, 2021; Ledgerwood & Shrout, 2011). We then validated the overall model and tested the mediation effects in the framework of structural equation modeling. Mplus 8.0 could simultaneously estimate all the coefficients required to test the H1–H4 hypotheses in the model. As the product of path coefficients used in estimating mediation effects did not follow a normal distribution, the bootstrap method was employed to estimate the mediation coefficients (Muthén & Muthén, 2017). As the bootstrap estimation for mediation coefficients can only be conducted using ML estimation, the latent serial mediation model was validated and tested using Mplus 8.0 with a maximum likelihood estimator, and the mediational paths were tested using 1000 bootstrapping samples with 95% bias-corrected confidence intervals.

The goodness-of-fit indices used in the model evaluation included: (1) Satorra–Bentler chi-square (for MLR estimator) or chi-square (for ML estimator); (2) root mean square error approximation (RMSEA); (3) standardized root mean square residual (SRMR); (4) TLI; and (5) comparative fit index (CFI). For the TLI and CFI, an acceptable fit cutoff was 0.900 and for a better fit it was 0.950. For the RMSEA, the criterion for a good fit was 0.060. A cutoff value of 0.080 for an adequate fit was often suggested for the SRMR. For the TLI and CFI, the bigger the value, the better the fit; the reverse was the case for the RMSEA, SRMR, and S-B chi-square. All indices can be combined to gauge the goodness of fit of the models (McNeish, 2024; Niemand & Mai, 2018).

## 3. Results

### 3.1. Common Method Bias

Data from the present study are mainly from self-reports and is thus susceptible to common method bias (Eid et al., 2016; Podsakoff et al., 2012). Harman’s single factor test was conducted by setting all items on a single common factor—the confirmatory factor analysis revealed that the model fitted the data poorly [S-B χ^2^(702) = 18,464.722, RMSEA = 0.119, CFI = 0.403, TLI = 0.370], suggesting that no significant bias exists in the present study.

### 3.2. Descriptive Statistics and Correlational Analysis

Descriptive statistics for each measure can be seen in the diagonal of Table 2. Correlations among average scores of each measure were all significant. Additionally, the latent correlations among the variables were also significant, and the correlational coefficients ranged from 0.238 to 0.810, which are suitable for further modeling.

### 3.3. Model Validation

**Figure 1:**
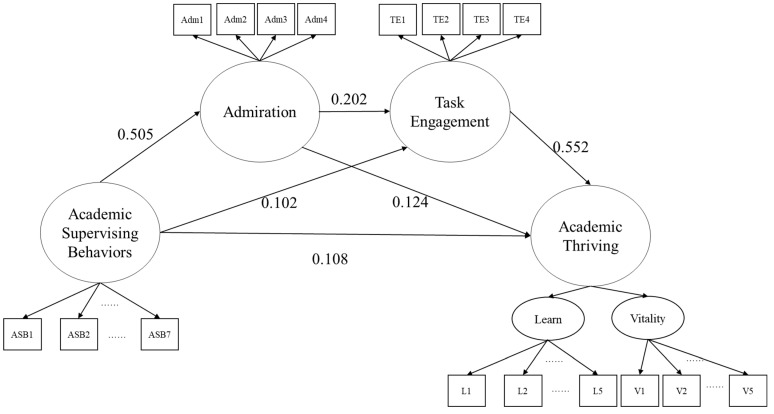
Latent serial-mediation model diagram. Path coefficients are all standardized. For the sake of simplicity, residuals are omitted and measurement models are simplified in the figure.

As in the past research, the two dimensions of academic thriving were closely correlated (0.844). We therefore modeled a second-order factor of academic thriving to account for the two dimensions. Based on the hypotheses, we tested the latent serial mediation model, as in Figure 1. As bootstrap estimation for mediation coefficients could only be conducted in models using the ML estimation, the model was estimated using the maximum likelihood estimator, and the fit indices suggested that the model fitted the data well: χ^2^(266) =1153.366, RMSEA = 0.043, CFI = 0.965, and TLI = 0.960. (Fit indices for the overall model using the MLR estimator: S-B χ^2^(266) = 930.571, RMSEA = 0.037, CFI = 0.965, TLI = 0.961).

### 3.4. Serial Mediating Effects

As in Figure 1 and Table 3, the total effect of academic supervising behaviors (ASBs) on academic thriving (AT) is significant. Furthermore, this total effect can be decomposed into three indirect paths. First, the indirect path of ASB-Engagement-AT was significant, as the bootstrapped confidence interval did not contain 0, with an estimated standardized path coefficient of 0.056 (19.788% of total effect). Second, the indirect path of ASB-Admiration-AT was also significant, as the bootstrapped confidence interval did not contain 0, with an estimated standardized path coefficient of 0.062 (21.908% of total effect). These results suggest that both admiration and task engagement can significantly mediate the effect of supervising behavior on graduate students’ academic thriving. Third, the serial mediational path of ASB-Admiration-Engagement-AT also reached significance, as the bootstrapped confidence interval did not contain 0, with an estimated standardized path coefficient of 0.056 (19.788% of total effect). The direct path was also significant, suggesting that the total effect was only partially mediated by the three indirect paths, accounting for 61.837% of the total effect.

## 4. Discussion

The present study examined the relation between supervisors’ academic supervising behaviors and graduate students’ academic thriving, with admiration for the supervisor and task engagement serving as mediators. This study found that supervising behaviors were positively associated with students’ academic thriving, and both admiration and task engagement significantly mediated this relationship.

### 4.1. Academic Supervising Behaviors and Academic Thriving

The present study found that graduate supervisors’ academic supervising behaviors can positively predict students’ academic thriving, suggesting that adequate supervision can facilitate positive performance and energetic mood for the graduate students. This finding supports H1 and adds to the growing literature on supervisor–student relationships (J. Han et al., 2022; Satinsky et al., 2021; Wu et al., 2023) by highlighting the importance of supervising behaviors. Previous work has mostly focused on the negative aspects of supervisor behaviors and their detrimental effects (Tenbrunsel et al., 2019; Yamada et al., 2014). The recent shift to the positive roles of graduate supervisors mostly examines supervisor support or leadership using subjective measurements (J. Han et al., 2022; Zhang et al., 2023). Less attention, however, has been paid to the type and frequency of supervising behaviors of graduate supervisors. The possible reasons for the effects of adequate supervising behaviors are threefold. First, sufficient supervising behaviors, such as providing help in setting research goals, monitoring the progress of research and study, providing resources, and providing timely feedback, are the basis for supervisors’ leadership or support. Second, the fact that supervisors are willing to frequently supervise or mentor the students would act as a powerful emotional signal, i.e., the students are valued by the supervisors as capable, though perhaps uninitiated, academic novices, rather than ones unworthy of their time. Such social verification can create psychological benefits (Hillman et al., 2022). Third, adequate supervision, instead of the hands-off supervising style, can substantially help most graduate students navigate various academic challenges. Some graduate students are well-prepared for the graduate experience themselves, but most are less familiar with the demands (e.g., timing, priorities, and standards), and timely and adequate guidance from their supervisor would ease their transition. In sum, adequate supervising behaviors can constitute a basis for the functioning of various roles of supervisors, can provide emotional reassurance, and can substantively help students to cope with challenges. These factors can contribute to the ultimate academic thriving of the students.

### 4.2. Mediation Role of Admiration and Academic Engagement

The present study further examined the mediating mechanism underlying the link between supervising behaviors and thriving. The findings reveal three mediational paths—the separate mediation of admiration and academic engagement, and the serial mediation of the two mediators. The findings provide support for H2–H4.

First, the mediating role of academic engagement between supervising behaviors and academic thriving is consistent with the previous research. Task focus and engagement were considered hallmarks of human agency (Spreitzer et al., 2005) and studies have shown that agentic work behaviors can predict thriving at work (Niessen et al., 2012; Paterson et al., 2014; Spreitzer & Porath, 2014; Yang et al., 2024). The previous research in work settings has documented that an important antecedent of agentic behaviors is unit climate (Spreitzer et al., 2005). Graduate supervisors’ supervising behavior can substantively and emotionally assist the graduate student, creating a supportive but directional environment, or “climate”, for the students, and spurring them to focus on and engage in academic tasks, which ultimately leads to academic thriving. The mediation effect presented in the present finding can add to the past literature that usually identifies both leader supervision (e.g., abusive supervision and authentic leadership in the past research) and task engagement/focus as simultaneous predictors of thriving (Liu et al., 2021). From the present study, it is shown that extrinsic academic supervising behaviors, if performed appropriately and sufficiently, can inspire a sustained, deeper engagement with the task at hand, creating an intrinsic dynamic (involving cognitive, emotional, as well as motivational aspects) that can culminate in academic thriving. This finding can lend further support to the socially embedded model of thriving at work (Istingtyas et al., 2025; Spreitzer et al., 2005), and shows that the agentic behaviors of graduate students (task engagement) can also lead to academic thriving in higher educational settings.

Second, the present study also found that the admiration for supervisors significantly mediated the link between supervising behaviors and thriving, while admiration and task engagement can further serially mediate the link. This finding aligns with the past research on the roles of positive emotions (e.g., Mikulincer & Shaver, 2020) and contributes to past models on thriving (Brown et al., 2017; Kern & Sun, 2020; Spreitzer et al., 2005), which do not formally specify the mechanism between the extrinsic vs. intrinsic aspects of the predictors for thriving. Specifically, admiration, as a positive emotion, can be seen as a missing motivational link between extrinsic supervision and individuals’ intrinsic state (self-involvement and task engagement). In higher educational settings, supervision-related behaviors serve as an interface where supervisors’ academic excellence or other virtues become more readily observable (e.g., by diligent and timely feedback, or astute comments), which elicit graduate students’ admiration for them (Lockwood & Kunda, 1999; Vianello et al., 2010). Graduate students’ admiration for supervisors was positively correlated with academic engagement in the present study, suggesting that admiration can also engender a tendency to emulate supervisors by becoming more engaged in academic tasks. Moreover, admiration for supervisors may also result in increased identification with academic values and beliefs represented by the supervisors, which can increase one’s academic engagement (Cesário & Chambel, 2017). Therefore, the present finding establishes a motivational route showing how supervisors’ behaviors elicit an emotional reaction (admiration) and then foster a motivational state that results in academic thriving. This is generally consistent with the self-determination theory (Deci et al., 1989) and theories on thriving. The finding also adds to the literature on emotion, motivation, and well-being in educational settings (Sosin et al., 2024; Wei et al., 2025). At the same time, admiration is itself motivating and can elicit experiences of vitality and self-efficacy (Schindler et al., 2015), which may consequently lead to thriving. This reasoning is also supported in the present study by the separate mediation effect of admiration between supervising behaviors and thriving.

In addition, from a motivational perspective (Deci et al., 2017), the serial mediation effect empirically demonstrated that graduate students could benefit from being extrinsically supervised and could gradually internalize the experience into a more intrinsically driven task engagement. Although one can argue that graduate students are already more intrinsically motivated than undergraduates and should be less reliant on supervision, given that they chose to pursue a higher level of study, the findings from the present study show that such an experience is indeed helpful. A developmental explanation could be that graduate students are still in the process of forming their own identity and cannot be construed as already highly self-determining; rather, the experience of being supervised may facilitate the identity-forming process. Another possibility is that each student may be motivated by an ensemble of different motives and mechanisms, and may be constantly shifting their motivational structure. Our findings can then be explained as a snapshot of the shift in action. For example, studies have increasingly shown that intrinsic motivation is not an unmitigated blessing or a silver bullet (Fishbach & Woolley, 2022; Kim et al., 2020; Yeager et al., 2014), and a combination of motivation with varying levels on the intrinsic–extrinsic spectrum can be more sustainable for effective adaptation. Future studies may more directly examine these possibilities.

In sum, the present study demonstrated that graduate supervisors can affect students’ academic thriving via adequate supervising behaviors, and this effect is further mediated by a socio-emotional pathway. Specifically, sufficient supervising behaviors would elicit student admiration, which inspires them to be more academically engaged and ultimately leads to increased thriving.

### 4.3. Implications and Limitations

This study has multiple implications. Theoretically, this finding extends the socially embedded model of thriving at work (Spreitzer et al., 2005) to the academic field. The role of supervisors has received relatively limited research, except in several studies (Brown et al., 2017; Paterson et al., 2014). Based on the present findings, a capable supervisor can clarify the meaning of a task, help set priorities, and supervise students as the task progresses, and instill an increased sense of identification with the task by providing timely and helpful feedback. These factors may contribute to greater agentic work behaviors in the supervisees, especially those lacking intrinsic motivation in the beginning (Kunter et al., 2008). Practically, for graduate education, aside from ethical requirements for the supervisors, the amount of supervising alone is important for the graduate student’s overall well-being; educators and educational policymakers may devise corresponding measures that guarantee that supervisors devote enough time and effort to supervise students and that an adequate supervision time is provided to each student.

The findings from this study also contribute to our understanding of supervisor–student relationships in Chinese contexts. Historically steeped in Confucianism, teachers as well as graduate supervisors are traditionally likened to one’s “academic father” (*Shifu*, or literally, teacher-father) in China (W. Liang et al., 2021; Y. Xu & Liu, 2023). Supervisors are expected to provide care and guidance, and act as custodians for students’ academic and personal development, a model that reflects Confucian patriarchal ideals. In return, the students are also expected to show obedience and unconditional devotion, in a manner that is consistent with Confucian filial piety (Y. Xu & Liu, 2023). As the modern academic system gradually takes root in China, a cultural mismatch occurs where some supervisors have started to refrain from actively supervising students but prefer to wait for students to use their own initiative (a more modern, egalitarian, but perhaps passive approach), while at the same time still enjoy the unconditional devotion from the students (a more traditional approach), all in the guise of egalitarianism and promoting students’ academic subjectivity. The lack of sufficient and effective supervision is thus a common complaint from graduate students in China (J. Han et al., 2022; Zhang et al., 2023). The findings from the present study shed light on the mechanisms underlying supervisors’ behaviors and their impacts on graduate students by showing how the frequency of supervising behaviors translate into student thriving via a socio-emotional pathway.

The present study also has some limitations. First, the cross-sectional nature of the present study precluded effective causal inference among the variables. Future studies may employ longitudinal designs, especially the data-intensive, ecological momentary assessment methodology, to better examine the causal relationship. Relatedly, future studies may also consider incorporating the supervisor-side and graduate–administrator-side sources of data (i.e., academic records and performance indices) as well as multiple potential confounding variables (e.g., demographic variables and health conditions) to facilitate a more comprehensive understanding of supervision and academic thriving. Moreover, the rules and modes of graduate education vary considerably across different countries, universities, disciplines, and even specific research teams. To add complexity to the diverse contexts, the subjective meaning of supervisory experiences and academic thriving also differ. Future studies that employ both quantitative and qualitative methods are needed to further explore its generalizability across different contexts. Furthermore, for graduate students, a few other antecedents to academic thriving warrant future research. For example, challenge stressors were found to predict thriving while hindrance stressors could not (Prem et al., 2017). Given that a certain amount of stress is inevitable during graduate education, a further examination of different types of stress could be beneficial to our understanding of thriving in graduate school. Lastly, due to the limitations of resources and feasibility, the present study employed convenient sampling from two research universities, rather than random sampling from different universities, which could restrict the generalizability of the results. It would be a fruitful avenue for future research to examine whether the present findings can be replicated in graduate students from different tiers of universities or research facilities.

## 5. Conclusions

Graduate students’ academic thriving is a positive marker of their adaptation in graduate school. The present study investigated the positive effect of supervising behaviors from supervisors by considering a socio-emotional mechanism based on the socially embedded model of thriving at work. We found that supervising behaviors could positively predict students’ academic thriving, and this effect could be separately and serially mediated by admiration and task engagement. We hope this finding inspires future studies that further examine possible antecedents to the well-being of graduate students.

## Figures and Tables

**Table 1:** Demographic information.

Categories	*N* (%)
Complete sample	1792 (100%)
Gender	
Male	937 (52.288%)
Female	855 (47.712%)
Type	
PhD candidates	135 (7.533%)
Masters students	1657 92.467%)
Major	
Humanities and social science	396 (22.098%)
STEM	1396 (77.902%)
Supervisor title	
Junior/Assistant Lecturer	2 (0.112%)
Lecturer/Assistant Professor	103 (5.748%)
Associate Professor	596 (33.259%)
Professor	1090 (60.826%)
Others	1 (0.056%)
Supervisor age	
30 yrs.	38 (2.121%)
31–40 yrs.	702 (39.174%)
41–50 yrs.	607 (33.873%)
51–60 yrs.	378 (21.094%)
Higher than 60 yrs.	67 (3.739%)

**Table 2:** Descriptive statistics and correlations.

	1	2	3	4	5
1. Task engagement	4.254 (1.276)				
2. Admiration	0.254 (0.220)	5.278 (1.196)			
3.Academic supervising behavior	0.204 (0.187)	0.505 (0.437)	3.343 (0.785)		
4. Academic thriving—learn	0.580 (0.526)	0.305 (0.204)	0.272 (0.224)	4.955 (1.027)	
5. Academic thriving—vitality	0.511 (0.448)	0.269 (0.232)	0.239 (0.194)	0.810 (0.695)	4.631 (1.030)

*Note*. All correlations are significant at the 0.001 level. Latent correlation coefficients (based on latent variables) are shown in the table, with manifest correlation coefficients (based on the average score) in parenthesis. Diagonals are means and standard deviations (in parenthesis).

**Table 3:** Mediation effect estimates.

	Standardized Effect Estimates	*SE*	*t*	*p*	Bootstrapped CI	
					Lower	Upper
Total effect	0.283	0.030	9.313	<0.001	0.224	0.343
ASB-Engagement-AT	0.056	0.018	3.068	0.002	0.020	0.093
ASB-Admiration-AT	0.062	0.017	3.715	<0.001	0.039	0.095
ASB-Admiration-Engagement-AT	0.056	0.011	5.144	<0.001	0.035	0.078
Total indirect effect	0.175	0.022	7.889	<0.001	0.132	0.219
Direct effect	0.108	0.032	3.357	<0.001	0.045	0.171

## Data Availability

The raw data supporting the conclusions of this article as well as the Mplus scripts are available by the first author on request.
